# Rehabilitation Management of the Charcot–Marie–Tooth Syndrome: A Systematic Review of the Literature

**DOI:** 10.1097/MD.0000000000003278

**Published:** 2016-04-29

**Authors:** Bruno Corrado, Gianluca Ciardi, Chiara Bargigli

**Affiliations:** From the Department of Public Health, University of Naples Federico II (BC, GC, CB), Naples, Italy.

## Abstract

The Charcot–Marie–Tooth disease (CMT) causes significant muscular deficits in the affected patients, restricts daily activities (ADL), and involves a severe disability. Although the conservative intervention is the only treatment for the disease, there is no scientific evidence so far on rehabilitation treatment. Objectives of the review are: research the best literary evidence so far on the rehabilitation treatment of CMT; critically analyze the outcome, to build an evidence-based work protocol.

A systematic review of the rehabilitation of a patient with CMT, including the results from the following databases: Pubmed, Medline, Embase, Pedro, Cinahl, Ebsco discovery. Criteria for inclusion: randomized/controlled studies, analytic studies, transversal studies on a cohort of at least 10 individuals; medium/long-term report of the results.

Eleven studies in total have been admitted to the final review phase; trials about physiotherapy CMT treatment (5), about orthosis treatment (6). Despite the wide range of outcomes and proposed interventions, the data points to the following: strength or endurance trainings improve functionality and ADLs of affected patients, while orthotic role is, at the moment, not completely clear.

Physiotherapy treatment is a useful tool to manage CMT; more studies on a larger number of cases are needed to define orthosis utility and to establish the gold standard of the treatment.

## INTRODUCTION

The term Charcot–Marie–Tooth (CMT) indicates a group of hereditary polyneuropathies, with both motor and sensorial manifestations, within the “hereditary motor and sensory neuropathy”.^1^ CMTs are the most common hereditary peripheral neuropathies in the world, with about 10 to 28 cases every 100,000 live births.^2^ The etiology of this disease is associated with alterations, not all of which are known in full, of homeotic genes involved in nerve formation;^1^ so far, mutations on chromosome 1 (CMT 1B), 17 (CMT1A), and X (CMTX) have been proved. The transmission model may be dominant autosomic, recessive or linked to the X chromosome;^1^ the dominant autosomic transmission is the most common in Europe.^2^

Traditionally, CMT pathophysiology has been categorized into 2 processes: a predominant demyelinating process resulting in low conduction velocities (CMT1) and a predominant axonal process resulting in low potential amplitudes (CMT2); many intermediate forms, however, have been discovered. CMT1, generally linked to mutations of the X chromosome, is divided into 4 subcategories (1A, 1B, 1C, 1D). The CMT2 (divided from 2A to 2P), on the other hand, is associated with an autosomal pattern, and features axonal degeneration; very rare is the CMT3 (dominant autosomal, with a hypomyelination pattern and axonal hypertrophy). Finally CMT 4, linked to an autosomal recessive pattern, is a group of axonal demyelinating neuropathies.

The disease starts in the first decade, with a distal involvement of the lower limbs, associated with hypotony and hyposthenia; the first symptoms are: tripping on the forefoot, ankle twisting, clumsiness when walking, calves cramps; in time, the stepping horse-like gait appears. Other evidence includes foot deformities, such as hammer-like toes and hollow feet. Poor knee control and frequent falls may lead he patient to need a wheelchair. As far as the upper limbs are concerned, fine motor skills decrease. Kyphoscoliosis appears in 10% of the cases.^3^

A sensorial deficit, at a superficial level, is characterized by pins and needles, burning pains, a needle-stick sensation in the distal parts of the limbs, while a deeper sensorial deficit alters the sense of position and can lead to lack of coordination and confidence when walking: sometimes, it develops into a magnetic gait on the ground and the supporting base may be extended.^4,5^ Among the complications of this disease are weakened respiratory and phonatory muscles, with an associated vocal chords paralysis.^6^

Progression is very slow and there may be long stand-by periods.^2^ Although CMT is destined to progressively worsen, functional outcomes may vary: from insignificant motor issues to complete limb atrophy, and in certain cases, the permanent need of a wheelchair. The instrumental diagnosis of the CMT includes an electrophysiological examination, showing the decrease of the motor conduction velocity (VCM) in the demyelination forms, as well as the potential motor and sensorial compound range in all forms. If collected data confirm the hypothesis, genetic research is carried out; a nerve biopsy will be only taken into consideration in uncertain cases.

Several therapeutic approaches have been attempted within CMT management: animal model showed usefulness of progesterone and ascorbic acid. The first was useful in promoting myelination in the peripheral nervous system; its action is transcriptional, and increases the genic expression of the proteins in peripheral myelin.^7^ On the other hand, ascorbic acid, typically used to promote collagen formation containing extracellular matrix, is now studied to trigger myelination in Schwann cells^8^ in case of demyelinating CMT.^9^ Human trials, however, had even adverse results.

Other conservative measures include the administration of drugs to control neuropathic pain, orthosis in case of mild/severe deficit in the foot muscles and physiotherapy. Several authors defined rehabilitation as a useful way to delay osteoarticular complications:^10^ nonetheless, there is currently absolute scientific uncertainty regarding both the work methods and the time and objectives of the rehabilitation management in a patient with CMT. Such a lack of knowledge means that the treatment provided may not be appropriate and that financial resources may be wasted. Objectives of this study:

1. To research the best evidence available in the literature to date regarding rehabilitation treatment of CMT, through a systematic review
2. To critically analyze the studies carried out, to build an evidence-based work protocol.

## METHODS

Since the study was a conducted systematic review, a permission from an ethic committee was not required.

### Sources and Key Words of the Search

A systematic review has been conducted, including the results of the following databases: Pubmed, Medline, Embase, Pedro, Cinahl, Ebsco discovery. In the last case, in particular, the research was conducted through the search engine of the University of Naples Federico II. To filter the evidence in the literature regarding the rehabilitation of the CMT, several key words have been used, as detailed in Table 1.

**TABLE 1:**
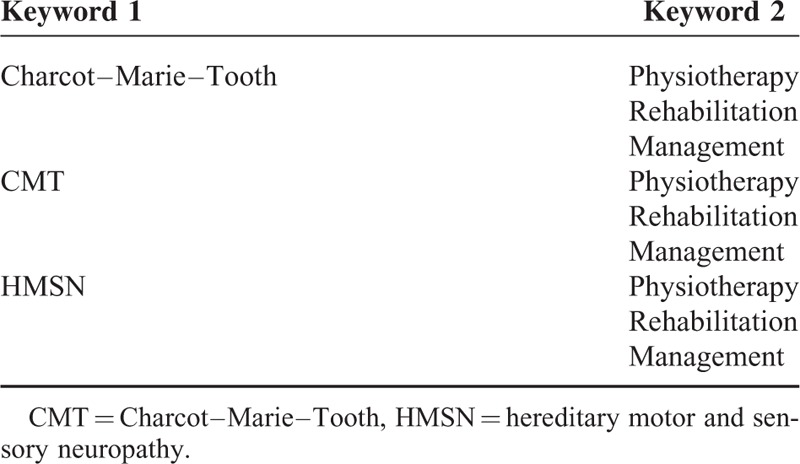
Keywords for the Search

### Criteria for Inclusion of the Studies

After defining the search queries, the criteria for inclusion were established, to verify the eligibility of the studies in the review:

1. Randomized/controlled studies, transversal analytical studies on a cohort of at least 10 individuals
2. Medium/long-term report of the results.

## RESULTS

**FIGURE 1:**
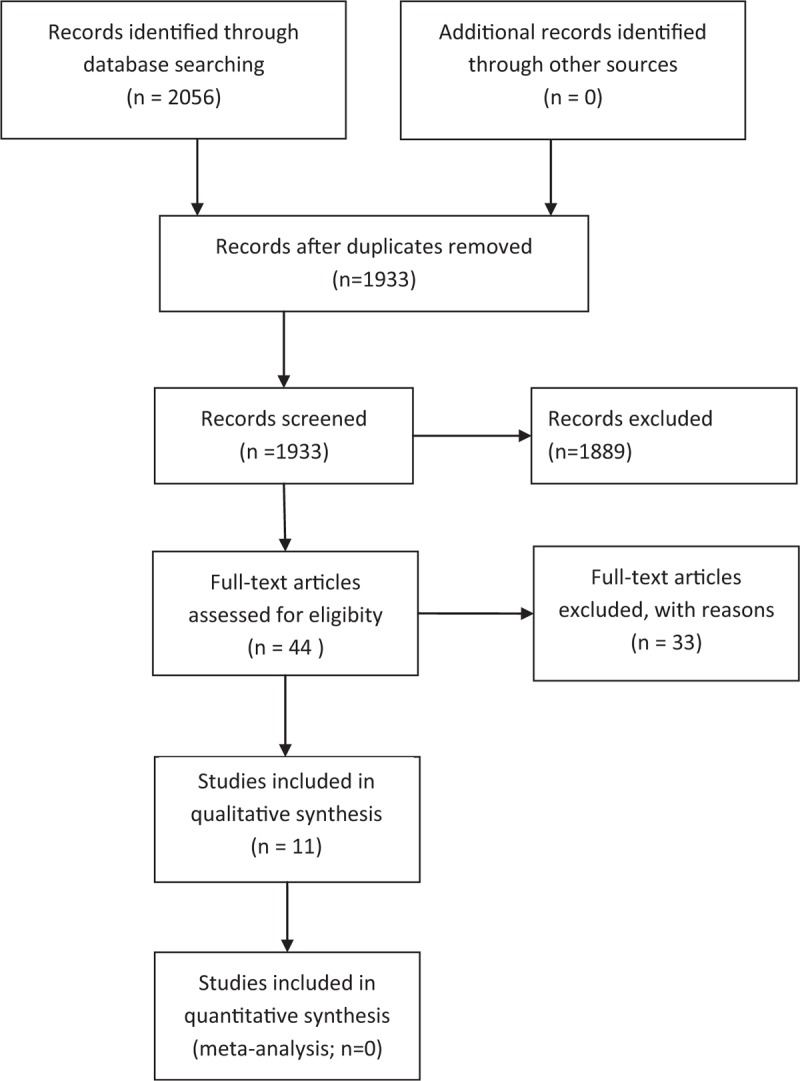
Flowchart of the study. The diagram reports the process of screening and choice applied to the results from all databases; starting by 2056 articles, only 11 were admitted at the last phase of the review.

A total of 2056 results emerged from this investigation, in the period between 1985 and 2015. The chart in Figure 1 provides a summary of the screening process of the evidence in the literature, while Table 2 details the results per database. The first screening excluded the following:

1. All studies registered in the databases more than once (n = 123)
2. Publications differing from the RCT/analytical studies: book extracts, conference proceedings (n = 1889).

**TABLE 2:**
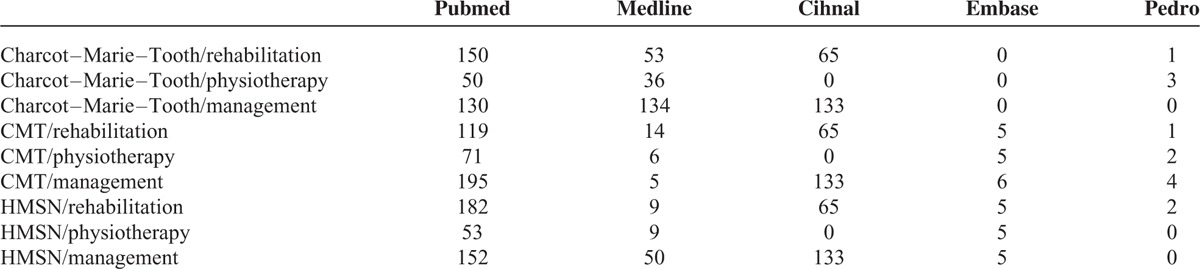
Number of Papers Among Different Databases

The 44 remaining studies were filtered according to the aforementioned inclusion criteria. The following were identified:

1. 7 RCT
2. 4 studies of cohort.

After this second phase, these studies have been admitted to the final phase of the review.

The evidence provided concerned 2 aspects of CMT rehabilitation: physiotherapy and orthotic treatment.

### Physiotherapy Treatment

Five studies in total, 4 of them experimental and 1 of cohort, met the inclusion criteria; Table 3 resumes the main characteristics of included trials.

**TABLE 3:**
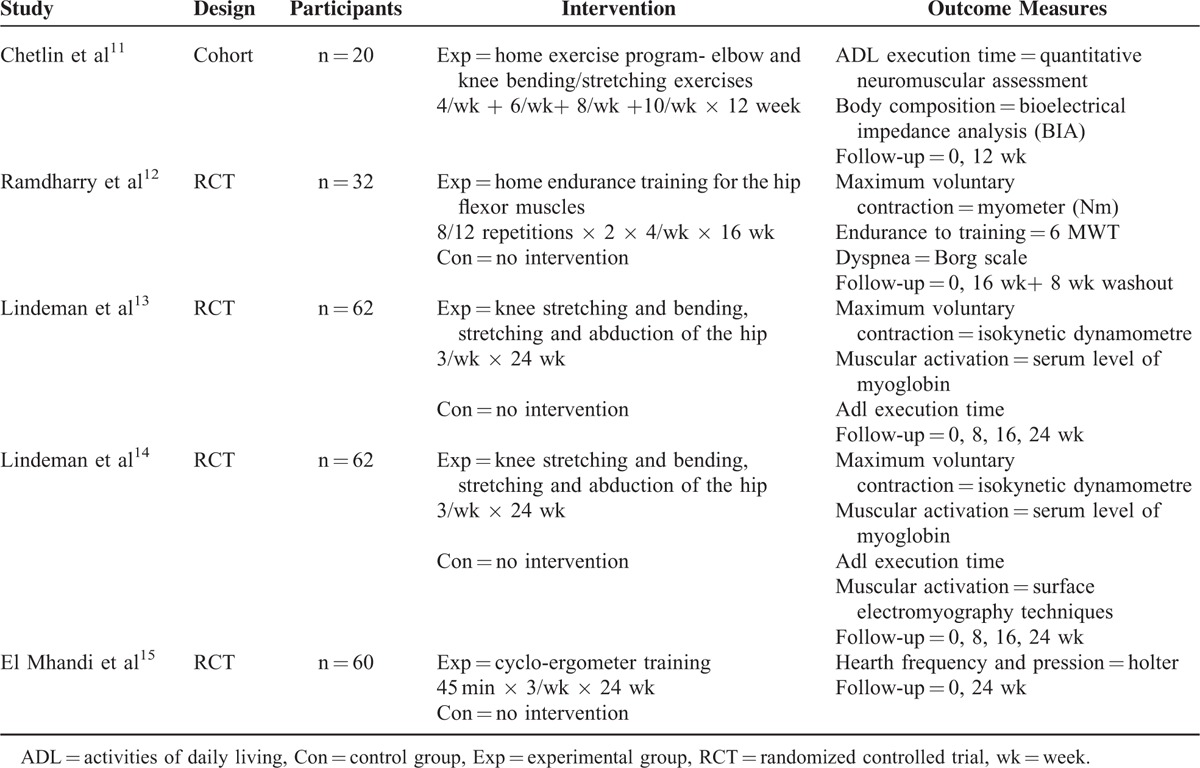
Summary of Included Studies—Physiotherapy Treatment

The first trial^11^ is aimed at assessing a home exercise program the patient will follow for 12 weeks, as well as drafting a series of endurance exercises based on the activities of daily living (ADL). The 20 patients received a detailed video on physiotherapy techniques to be performed at home. The results of the study showed an increase of the muscular strength, and a shorter ADL execution time following the therapy recommended to both groups.

Likewise, the Ramdharry study^12^ investigated the effects of a home endurance training lasting 16 weeks, aimed at the recovery of the hip flexor muscles. The 32 participants were divided into training and control group; the study included 16 weeks of intervention and 8 of rest and periodic checks. The patients were monitored through an exercise diary, weekly calls, and monthly check-ups. The authors registered an increase of the flexors force in the left hip, whereas no significant improvement has been registered in the draft resistance and walking speed.

The third study^13^ analyzed was aimed at determining whether muscular strength training is effective, on a short-term basis, to improve impairments (loss of strength), disability (decrease of functionality), and handicaps (well-being) of patients with a neuromuscular pathology. Sixty-two patients were selected, 33 with myotonic dystrophy and 29 with HSMN (CMT), and were divided into a training group and a control group. The results showed a substantial neutrality of the effects in patients with myotonic dystrophy, whereas patients with CMT showed a moderate recovery of leg strength, as well as a better ADL performance.

In his study, Lindeman et al,^14^ who were also involved in the previous trial, repeated the same study and compared patients with polyneuropathy and patients with myotonic dystrophy.

Studied population, suggested exercises, and stages recommended were the same as the previous study, as well as the progression and duration of the follow-up. As for the outcome, the authors also introduced surface electromyography techniques (SEGM) to assess the activation of each muscular ventral part during isometric contractions. The results show an increase of the isometric force in the muscular ventral parts of the quadriceps; this increase was initially due to neural factors, then to hypertrophy from exercising.

The last study analyzed, conducted by El Mhandi et al,^15^ sought to investigate the effects of a rehabilitation program on the variation of the cardiac frequency in patients with CMT.

The 16 participants were divided into 2 groups: affected by CMT and healthy ones. The program included a cycle-ergometer training, 3 times a week and for 24 weeks; this training led to an improvement of the cardiac frequency, especially at night.

### Orthosis Treatment

Six studies, 3 RCT and 3 of cohort, were admitted to the review; Table 4 details main trials characteristics.

**TABLE 4:**
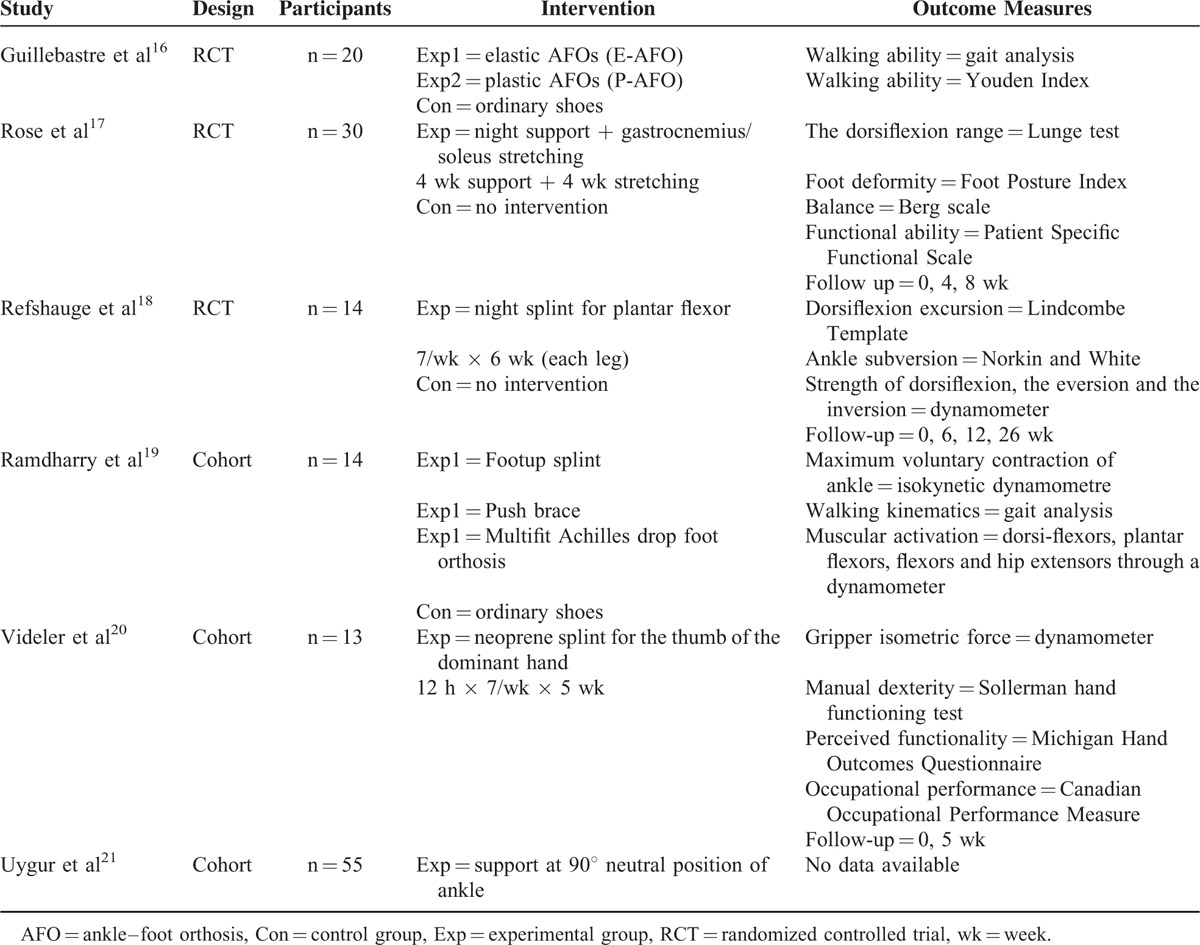
Summary of Included Studies—Orthosis Treatment

The first study^16^ verified the hypothesis that ankle-foot orthosis (AFOs) improve posture and walking control in CMT. The authors recruited 26 patients for this purpose, and divided them into 3 groups, wearing, respectively, ordinary shoes (control), plastic AFOs (P-AFO), and elastic AFOs (E-AFO). Gait analysis and Youden Index showed plastic AFOs to improve both the posture control and walking, whereas elastic orthosis influenced the dynamic control of deambulation. The results showed that AFO prescription is relevant for improving walking balance and performance.

Rose study^17^ is aimed at verifying whether applying a night support over 4 weeks, as well as undertaking an equal period of stretching of the gastrocnemius and soleus muscles, can lead to an improvement in the ankle dorsi-flexion range. Thirty children and teenagers affected by CMT took part in the study, divided into control and intervention group. The authors report that night orthosis improved the dorsiflexion of the ankle by 4 degrees; after 4 weeks of stretching, the dorsiflexion improved by 3 degrees than the original range.

The Refshauge study^18^ investigated the effect of a night splint for plantar flexor's extension on the articulation range of the ankle in a patient affected by CMT. There were 14 participants, aged between 7 and 30. A support was applied in a condition of maximum dorsiflexion on 1 leg overnight and for 6 weeks; subsequently, the same treatment was applied on the other leg. The results showed that, by wearing the night splint, there were no significant differences in the range of motion (ROM) or strength of inversion/eversion movements.

The fourth study^19^ investigated the effect of the different AFOs on the distal control of the leg and proximal compensatory actions. The 14 participants were tested while wearing 3 types of AFOs or normal shoes. The first AFO was a “Footup splint,” a cuff worn around the ankle with a detachable elastic strap and a plastic flange at 1 end to tie into the laces of a shoe. The second, instead, was a “Push brace,” designed as a support for ankle instability following ligament injury. The main brace was made of preformed foam covering the medial, posterior, and lateral aspects of the ankle and extending under the calcaneum. The Multifit Achilles drop foot orthosis, finally, is a variant of the posterior leaf AFO made of plastic polymer with a half foot plate, a cut-out heel section and an adjustable back stem that extends to the calf. No significant differences were registered in the speed and step length of patients wearing AFOs and patients wearing standard shoes. The authors pointed out a significant influence of the AFOs on hip flexion at the oscillating stage of the step, and on the dorsiflexion of the ankle; this prevented the foot from falling and reduced the tripping frequency.

The Videler study^20^ assessed the effectiveness and tolerance level of a splint for thumb opposition on manual dexterity, functionality of the upper limbs, and occupational performance. Thirteen patients were provided with a neoprene splint for the thumb of the dominant hand, and trained to use it as long as possible, over a 5-week period. The results show that splinting significantly increases functionality in daily activities as well as occupational performance.

The Uygur study^21^ has been conducted on 55 pediatric patients. The children had to wear a support to ensure a neutral position and a 90° ankle overnight; no outcome or follow-up data were provided in this case. The conclusion was that children should avoid any risk related to a non-necessary orthosis treatment.

## DISCUSSION

This review complies with the document issued by the PRISMA group regarding meta-analysis drafting and systematic reviews.^22^ In the following section, we will analyze the possibilities for comparison of detailed evidence.

As for the physiotherapy treatment of CMT, attention must be drawn to the following aspects:

1. Physiotherapy is similar in some of the evidence: the muscular training focused on the strength^13,14^ or endurance.^11,12^
2. Despite the verisimilitude of the objectives, a wide range of methods were used to measure the outcome of the evidence: muscular strength, the only common measurement, was evaluated through different methods (QMA, 1RM, myometre), so it does not allow any comparison.
3. As for the results, all evidence points to the effectiveness of physiotherapy in CMT, as it is associated with an increase of the strength.^11–14^ The muscles involved are different in different trials: the muscles of the arm,^11^ the pelvic girdle and thigh (hip flexors and quadriceps),^12–14^ and the knee.^11,13,14^ The evidence agrees on the improvement in timing of execution of ADLs,^11,13^ and only 1 case did rehabilitation prove ineffective with regard to walking speed;^12^ in another isolated case, physiotherapy proved effective as regard the modulation of the parasympathetic system.^15^

These findings only partially tally with the data present in the literature; a recent document by Cochrane group^23^ shows that training programs for CMT increase the deambulation speed, but do not significantly increase ADLs. On the other hand, comparing these results with another systematic review,^24^ they match: a clear improvement in strength is shown, as well as absence of side effects after physiotherapy, as postulated in the trials considered.^11^

As for the orthosis treatment of CMT, and regarding the possibility of comparison, the following can be observed:

1. As for the intervention suggested, many studies^16–19^ adopted a support on the lower limbs, while just 1 study suggested a device on the upper limbs;^20^ only 1^21^ discussed the possibility of both.
2. The outcome varies considerably from 1 study to another: articular excursion,^17,18^ strength,^18–20^ balance,^17^ walking kinematics,^16,19^ restrictions on functionality.^17,20^ For ankle excursion in dorsiflexion, which is the most common outcome, the measurement method varies: questionnaires,^20^ dynamometric tests,^18–20^ functional tests.^16–18,20^
3. In addition, different studies showed different results; if a little evidence is found in the increase of the ankle dorsiflexion,^17,19^ each study also reports an improvement in balance,^16^ a better walking performance,^16^ time reduction when walking small distances (10 m),^17^ a decrease in hip flexion and in tripping.^19^ Just 1 trial^18^ failed to find significant differences in ROM and dorsiflexion strength. As for the upper limbs, the authors of the study^20^ report an improvement in daily life functionality, in occupational performance and satisfaction.

Comparing these results with the reviews in the literature^25–27^ opposite views can be observed on the orthosis treatment of patient with CMT: a paper by the Cochrane group,^25^ unlike 2 trials,^17,19^ indicated that there is no evidence of significant benefits for the articular excursion of the ankle. On the other hand, another paper^26^ focuses on the pediatric population with CMT, and demonstrates that orthotics are suitable in case of hollow feet, foot ache, and/or mild balance alteration, whereas AFOs are suitable for falling feet, general weakness of the feet muscles, severe balance alterations, and/or deambulation difficulties. This conclusion partially tallies with the present paper, particularly for the prescription of AFOs.^16,19^ Another review,^27^ in accordance with the results of one of the trials considered,^21^ suggests customization of the product (shoes, orthotics, orthosis) provided to the CMT patient.

## RISK OF BIAS

The systematic revisions of the scientific literature are exposed to a double risk of systematic errors: bias on each study and comparison bias. Given the fact a comparison bias is impossible, and given the variety of the outcomes, the Downs and Black checklist was used, in the attempt to identify all possible bias.^28^ Such a tool allows effectively assessing the methodology quality of the random controlled and nonrandom studies, through a score from 0 to 31. For the present review, the scores vary from 19 to 23, with a predominance of the 22 to 23 score. It is so possible to say the studies included in the review have a medium-high scientific accuracy. Table 5 shows the scores obtained by each study according to the checklist.

**TABLE 5:**
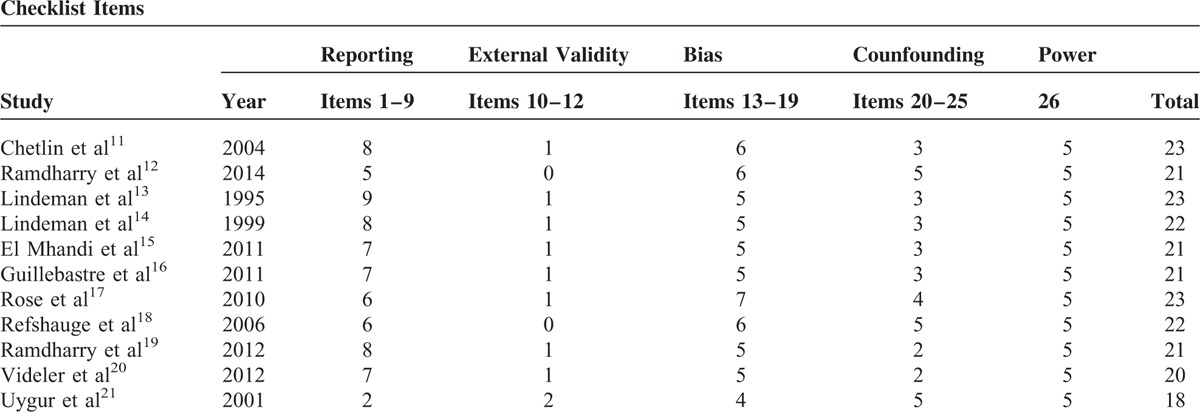
Downs & Black Checklist for the Risk of Bias

Several considerations about physiotherapy management of CMT derived from this review:

1. With regard to rehabilitation, this review proved to be a mainstay in the therapeutic approach. In particular, individual training programs contributed to the improvement of muscular strength in both upper and lower limb in various trials.
2. Orthosis role in CMT management has to be further studied: isolated data showed it as associated with a significant improvement of the ROM in the ankle dorsiflexion, thus allowing a better walking kinematic. These conclusions, however, cannot be considered evidence based. Also hand orthosis must be studied and tested more in depth, as just 1 RCT showed its benefits.
3. Unfortunately, it must be stressed that the scientific literature available to date focuses only on the purely clinical aspects of CMT, neglecting the useful and innovative aspects of the rehabilitation; due to the lack of data and to the limited comparison between trials, an evidence-based protocol cannot be, to date, established. The hope for future is that new clinical trials will be conducted on the rehabilitation of patients with CMT, including more case studies, and defining the gold standards in the rehabilitation management of such patients.
